# Maternal body burdens of PCDD/Fs and PBDEs are associated with maternal serum levels of thyroid hormones in early pregnancy: a cross-sectional study

**DOI:** 10.1186/s12940-016-0139-7

**Published:** 2016-04-26

**Authors:** Sanna Lignell, Marie Aune, Per Ola Darnerud, Mats Stridsberg, Annika Hanberg, Susanna C Larsson, Anders Glynn

**Affiliations:** Risk Benefit Assessment Department, National Food Agency, Box 622, SE-751 26 Uppsala, Sweden; Chemistry Department, National Food Agency, Box 622, SE-751 26 Uppsala, Sweden; Department of Medical Sciences, Uppsala University, SE-751 85 Uppsala, Sweden; Institute of Environmental Medicine, Karolinska Institutet, Box 210, SE-171 77 Stockholm, Sweden

**Keywords:** Thyroid hormone, T4, T3, TSH, Dioxins, PCBs, PBDEs

## Abstract

**Background:**

Thyroid hormones (THs) regulate many biological functions in the human body and are essential for normal brain development. Epidemiological studies have observed diverging associations between halogenated persistent organic pollutant (POP) exposure and concentrations of THs in pregnant women and their infants. We investigated whether background exposure to polybrominated diphenyl ethers (PBDEs) is related to TH status in a Swedish population of pregnant women and their infants. Furthermore, we examined associations between polychlorinated dibenzo-*p*-dioxins/dibenzofurans (PCDD/Fs) and polychlorinated biphenyls (PCBs) and TH status in early pregnancy as an extension of an earlier study focusing on late pregnancy TH status.

**Methods:**

Free thyroxine (T4), total triiodo-thyronine (T3) and thyroid stimulating hormone (TSH) were analysed in serum from first-time mothers (*N* = 220-281) in the first and third trimester, and in infants (*N* = 115-150) 3 weeks and 3 months after delivery. Antibodies to thyroid peroxidase (anti-TPO) (*N* = 260) were measured in maternal third trimester serum. Maternal body burdens of PCBs (N = 281) were estimated from serum lipid PCB concentrations in late pregnancy, and PCDD/F (*N* = 97) and PBDE (*N* = 186) body burdens were estimated from concentrations in mother’s milk lipids 3 weeks after delivery. Linear regression models allowed for covariate adjustment of the associations between ln-transformed POP body burdens and concentrations of TH and anti-TPO.

**Results:**

Maternal body burden of BDE-153 was inversely associated with first trimester total T3, otherwise no associations between PBDEs and first and second trimester THs were observed. No associations were found between maternal PBDE body burdens and infant THs. Maternal body burden of PCDD/Fs were inversely associated with first trimester total T3. No associations were observed between PCBs and first trimester THs. Third trimester anti-TPO was not associated with maternal PCBs, PCDD/Fs and PBDEs.

**Conclusions:**

Our results suggest that maternal PCDD/F and BDE-153 body burdens influence maternal TH status in early pregnancy, which is a critical period when maternal TH status influences fetal development.

## Background

Thyroid hormones (THs) regulate a wide array of biological functions in the human body, including metabolism and development. THs are essential for normal brain development, particularly during the fetal period [1]. Severe maternal and congenital hypothyroidism is associated with impaired child neurodevelopment, and hypothyroid fetuses suffer various postnatal disorders including mental retardation, deafness and spasticity [2]. Even relatively minor reductions in maternal free thyroxine (T4) blood concentrations in early pregnancy, with normal thyroid stimulating hormone (TSH) concentrations, have been associated with impaired cognitive development among children [2]. However, THs play many other roles during pregnancy, for instance by local action of THs on female reproductive organs and embryo during implantation [3, 4]. Moreover, THs are essential for normal fetal growth [5]. It has been suggested that any alteration in maternal and fetal thyroid function has implications for health of both the fetus and mother [3, 5].

Several halogenated persistent organic pollutants (POPs) have chemical structures similar to THs, and there are many studies of POP-induced TH disruption in laboratory animals. Polychlorinated dibenzo-p-dioxins and polychlorinated dibenzofurans (PCDD/Fs), polychlorinated biphenyls (PCBs) and polybrominated diphenyl ethers (PBDEs) reduce the concentrations of circulating free and total thyroxine (T4) in animals. In some studies PCBs and PCDD/Fs have increased the concentrations of thyroid stimulating hormone (TSH) [4, 6], suggesting a feedback response to stimulate TH production.

Epidemiological studies on associations between background exposure of pregnant women and their infants to PCDD/Fs and PCBs and blood concentrations of THs indicate inverse associations with triiodothyronine (T3) or T4 and in some cases positive associations with TSH [7–11]. Conflicting results or absence of significant associations have also been reported [12–15]. Studies on associations between exposure to PBDEs and TH concentrations in pregnant women and infants are still limited. The results are inconclusive and indicate inverse or positive associations, as well as no statistically significant associations [14, 16–22].

The aim of the present study was to investigate associations between background maternal body burdens of PCBs and PCDD/Fs during pregnancy and first trimester maternal TH concentrations in a cohort of Swedish mother-infant pairs (POPUP cohort; Persistent Organic Pollutants in Uppsala Primiparas). Many previous studies have measured third trimester THs [8, 9, 23]. In the current study we considered it relevant to study maternal TH concentrations in early pregnancy, since the fetus is dependent on maternal THs during this period for normal development [5]. Since we previously investigated associations between maternal PCB/PCDD/F body burdens and TH in late pregnancy in the same women [24], we now also have the possibility to determine if associations persist from the first to the third trimester, or if associations differ between the two time-points. In the earlier study we found an inverse association between maternal body burdens of PCDD/F and total T3 in late pregnancy. No significant associations were found for PCBs [24].

In the POPUP cohort we have previously observed an inverse association between maternal body burden of sumPBDE (BDE-47, 99-, −100 and −153) and birth weight [25]. Maternal TH status during pregnancy is an important determining factor for fetal growth [5], and PBDE-induced alterations in TH status could be a mechanism behind our PBDE/birth weight observation [25]. In the current study we therefore examined associations between maternal body burdens of tetra- to hexa-brominated PBDEs and maternal first and third trimester TH concentrations, in an effort to shed a light on possible effects of background PBDE exposure on maternal thyroid function. PBDEs were not investigated in our previous study [24]. In the current study we also investigated associations between maternal PBDE and THs in 3 weeks and 3 months old infants. In the previous study, not including PBDEs, no associations were observed between maternal PCDD/Fs and PCBs and THs in 3-weeks and 3-months old infants [24].

Finally, in the present study we investigated associations between maternal PCDD/F, PCB and PBDE body burdens and maternal third trimester concentrations of antibodies to thyroid peroxidase (anti-TPO), a useful biomarker of thyroid dysfunction. Only a few studies have addressed the possibility that POPs may cause thyroid dysfunction through anti-TPO. A study in a highly PCB polluted area in Slovakia showed a positive association between PCB body burdens in adults and anti-TPO [26]. In an adult population with background PCB exposure in Italy no significant association was found [27].

## Methods

### Study population, sampling and data collection

The POPUP study is an on-going study that includes randomly selected first-time mothers from Uppsala County, Sweden [28, 29]. During the first years of the study (1996–1999), 325 pregnant women were recruited [30]. Participants donated non-fasting blood samples in early (first trimester, week 6–12) and late (third trimester, week 32–34) pregnancy. After delivery, mother’s milk was donated by 204 women and blood was sampled from 160 infants at 3 weeks and from 138 infants at 3 months of age. The milk sampling was conducted at home during the third week after delivery.

The present study of THs includes 307 mother-infant pairs recruited in 1996–99, of which 196 donated mother’s milk. Due to limited blood serum volumes in the present study, first trimester THs were analysed in 220 maternal blood serum samples. TH data from the third trimester, and from infants 3 weeks (*N* = 150) and 3 months (*N* = 115) after delivery, were taken from the previous study [24]. Concentrations of anti-TPO were in the present study determined in third trimester maternal serum (*N* = 260). PCB concentrations in maternal third trimester serum were obtained from the previous study [24], as well as mother’s milk data on PCDD/Fs. PBDEs were analysed in mother’s milk in the present study. Data on maternal and child characteristics were obtained from interviews, questionnaires and from the Swedish Medical Birth Register at the National Board of Health and Welfare [25, 30]. The study was approved by the local ethics committee of Uppsala University (reference number 96114 and 2004:M-177), and participants gave their informed consent prior to the inclusion in the study.

### Chemical analysis

#### Thyroid hormones

Non-fasting serum concentrations of total T3, free T4 and TSH were measured on an automatic immunoassay system (Autodelfia, Wallac Oy, Turku, Finland). In the present study THs from the first trimester were analysed in addition to the third trimester TH analysis from the previous study [24], using the same analytical method. Moreover, total T3 free T4 and TSH data for infants (3 weeks and 3 months) were available from the previous study [24], also using the same analytical methods as in the present study. In the present study, measurements of anti-TPO in third trimester serum were performed on an automatic immunology analyser (Cobas E601, Roche Diagnostics, Basel, Switzerland). The total assay variation in the individual assays was less than 5 %. All analyses were performed at the routine laboratory of the Department of Clinical Chemistry at the University Hospital in Uppsala. The laboratory is certified by a Swedish government authority (Swedac).

#### POP in blood serum and mother’s milk

Blood serum samples collected in the third trimester were in the previous study analysed for di-*ortho* substituted PCB congeners (PCB 138, 153 and 180) and mono-*ortho* substituted PCB congeners (PCB 105, 118, 156 and 167) (*N* = 281) [24]. A randomly selected number of mother’s milk samples were at the same time analysed for seventeen 2,3,7,8-substituted PCDD/Fs (*N* = 97) [24]. In addition, in the present study four PBDE-congeners (BDE-47, BDE-99, BDE-100, BDE-153) (*N* = 186) were analysed in mother’s milk.

PCBs in blood serum and PBDEs in mother’s milk were analysed at the Swedish National Food Agency (NFA) according to previously described methods [29, 30]. After extraction and clean-up, the quantification of PCBs was performed on a gas chromatograph equipped with dual capillary columns and electron capture detectors (^63^Ni) [28]. PBDEs were analysed by gas chromatography/low resolution mass spectroscopy/electron capture negative ionization and detected by single ion monitoring technique [28]. All samples were fortified with internal standards prior to extraction to correct for analytical losses and to ensure quality control. The limit of quantification (LOQ) varied between 2 and 4 ng/g lipid for PCBs and between 0.1 and 1.3 ng/g lipid for PBDE congeners.

PCDD/Fs in mother’s milk was analysed at the National Institute of Public Health and the Environment (RIVM), the Netherlands, using a method earlier described [28, 31]. Briefly, samples were analysed according to validated procedures consisting of milk extraction, several clean-up steps including active carbon chromatography, and identification and quantification of the analytes using gas chromatography with high-resolution mass spectrometry with isotope dilution technique. LOQ varied between 0.1 and 1.0 pg/g lipids for the individual PCDD/F congeners.

The lipid content of each mother’s milk and blood serum sample was determined gravimetrically. A number of control samples were analysed together with the samples to verify the accuracy and precision of measurements. The laboratories at the NFA and RIVM have successfully participated in several international proficiency tests.

### Data analysis

#### Estimation of exposure

Lipid-adjusted maternal blood serum concentrations (PCBs) or mother’s milk concentrations (PCDD/Fs, PBDEs) were used to estimate maternal body burdens during pregnancy as well as fetal exposure. When the concentrations were below the LOQ, half of LOQ was taken as an estimated value. POPs were grouped into di-*ortho* PCBs (sum of PCB 138, 153 and 180), mono-*ortho* PCB TEQ (toxic equivalents) (sum of TEQs for PCB 105, 118, 156 and 167), PCDD/F TEQ (sum of TEQs for 17 PCDD/F congeners), and tetra-pentaPBDE (sum of BDE-47, −99 and −100). The hexa-brominated BDE-153 was treated separately in the statistical analyses since the tetra-pentaPBDEs are strongly correlated with each other, but not with BDE-153 in mother’s milk in the POPUP cohort [29]. Moreover, BDE-153 seems to deviate from the lower brominated PBDEs with regards to toxicokinetics [29]. For the dioxin-like PCBs and PCDD/Fs, the WHO 2005 toxic equivalency factor (TEF) system was used when the concentrations of the substances were combined [32].

#### Statistical models

In the current study we investigated associations between maternal body burdens of PCBs (maternal serum), PCDD/Fs and PBDEs (mother’s milk) and first trimester TH concentrations. In addition, associations between maternal PBDE body burdens and maternal TH concentrations in the third trimester, as well as infant TH concentrations 3 weeks and 3 months after delivery, were studied. Finally, associations between all analysed POPs and maternal third trimester concentrations of anti-TPO were assessed.

Statistical analyses were performed using MINITAB 15^®^ Statistical Software for Windows. A *p*-value ≤0.05 was considered statistically significant in all statistical tests. We used linear regression models to investigate associations between exposure to POPs and serum TH and anti-TPO concentrations. To reduce the effect of outliers, and to get normally distributed exposure data, all POP concentrations were ln-transformed. Total T3, free T4 and TSH concentrations were approximately normally distributed (Shapiro-Wilk W-test, *W* = 0.86-0.99). However, anti-TPO concentrations were right-skewed and were thus ln-transformed. After a crude analysis, possible confounders considered for inclusion in the maternal TH models were maternal age, country of birth, pre-pregnancy BMI, education, smoking during pregnancy, alcohol consumption during pregnancy, maternal weight gain during pregnancy and season of sampling. In addition, variables considered for inclusion in infant TH models were gestational length, mode of delivery, gender of the infant, birth weight, sampling day (number of days after delivery) and infant weight at 3 months. Associations between the possible confounders and outcomes were first investigated in bivariate models (Pearson correlation analysis or one-way analysis of variance (ANOVA)). Covariates associated with any of the maternal or infant outcomes (*p* ≤ 0.05) were included in the final models. Covariates were included as continuous variables or categorized as shown in Table 1. Season was categorized as winter (December-February), spring (March-May), summer (June-August) and autumn (September-November). The number of samples analysed for the different POPs varied, and analyses restricted to individuals with complete datasets were performed to investigate if the results were robust to changes in number of study participants. The fit of the regression models was checked by plotting the residuals around the fitted line. The robustness of the results was also evaluated by a sensitivity analysis where outliers with a standardized residual ≥3 were excluded from the regression models.

**Table 1:** Characteristics of the study participants, i.e. 307 mother-infant pairs recruited in 1996-1999

Characteristic	N	Median (Range)
Maternal age (years)	307	28 (18–41)
Maternal pre-pregnancy BMI (kg/m^2^)	307	23 (18–44)
Maternal weight gain during pregnancy (%)^a^	304	22 (3.9–46)
Gestational length (days)^b^	138	280 (249–300)
Birth weight of the child (g)^b^	150	3510 (2430–5000)
Age of infant at 3-weeks-sampling (days)^b^	148	24 (17–38)
Weight of the infant at 3 months^c^	109	6180 (4350–8600)
Age of infant at 3-months-sampling (days)^c^	115	91 (75–123)
Maternal serum TH concentrations, early pregnancy		
TSH (mIU/L)	220	1.3 (0.002–7.5)
Total T3 (nmol/L)	219	2.4 (0.57–3.7)
Free T4 (pmol/L)	220	11 (7.8–20)
Maternal serum TH concentrations, late pregnancy		
TSH (mIU/L)	281	1.4 (0.06–6.7)
Total T3 (nmol/L)	281	2.5 (1.1–3.8)
Free T4 (pmol/L)	281	8.3 (5.7–13)
anti-TPO^d^ (kIU/L)	260	8.6 (5.5–134)
Infant serum TH concentrations, 3 weeks of age		
TSH (mIU/L)	148	2.4 (0.54–6.4)
Total T3 (nmol/L)	145	2.8 (1.4–4.2)
Free T4 (pmol/L)	150	17 (12–23)
Infant serum TH concentrations, 3 months of age		
TSH (mIU/L)	115	1.8 (0.77–4.8)
Total T3 (nmol/L)	115	3.0 (1.7–4.2)
Free T4 (pmol/L)	114	15 (10–22)
	N	%
Country of birth		
Nordic country	291	95
Non-Nordic country	16	5
Years of education		
≤4 years of high school	144	49
1–3 years of higher education	72	24
>3 years of higher education	79	27
Smoking habits during pregnancy (smoker/non-smoker)	58/194	19/81
Alcohol during pregnancy (no/yes)	254/52	83/17
Mode of delivery (vaginal/caesarean)^b^	120/18	87/13
Sex of the child (male/female)^b^	83/67	55/45

^a^(weight gain during pregnancy/weight before pregnancy)*100

^b^Among 150 infants with analysed THs in serum at 3 weeks of age

^c^Among 115 infants with analysed THs in serum at 3 months

^d^Thyroid peroxidase antibodies

## Results

Characteristics of the participating women and children as well as blood serum concentrations of THs are given in Table 1. PCB concentrations in maternal blood serum and breast milk PCDD/F and PBDE concentrations are presented in Table 2. In blood serum, di-*ortho* PCBs was the substance group with the highest median concentration (128 ng/g lipid). The median concentration of PCDD/F TEQ in mother’s milk was 7.9 pg/g lipid. BDE-47 was the dominating PBDE-congener with a median mother’s milk concentration of 1.6 ng/g lipid.

**Table 2:** POP concentrations in serum and mother’s milk among mothers with measured maternal and infant thyroid hormone concentrations

	Maternal		Infant	
	*N*	Median (range)	*N*	Median (range)
Serum^a^				
di-*ortho* PCB (ng/g lipid)	281	128 (28–367)		
mono-*ortho* PCB TEQ (pg/g lipid)	281	0.56 (0.2–3.6)		
Breast milk^b^				
PCDD/F TEQ (pg/g lipid)	91	7.9 (3.3–16)		
tetra-pentaPBDE (ng/g lipid)	166	2.3 (0.46–31)	130	2.4 (0.54–31)
BDE-153 (ng/g lipid)	166	0.48 (<0.2–4.3)	130	0.49 (<0.2–2.2)

^a^di-*ortho* PCB = sum of PCB 138, 153 and 180; mono-*ortho* PCB TEQ = sum of TEQs for PCB 105, 118, 156 and 167 [32]

^b^PCDD/F TEQ = sum of TEQs for the 17 congeners with WHO TEFs [32]; tetra-pentaPBDE = sum of BDE-47, −99 and −100

In the crude analysis, there were significant inverse associations between di-*ortho* PCB and mono-*ortho* PCB TEQ concentrations in serum lipids (third trimester) and first trimester maternal serum concentrations of total T3 (Table 3). Similar associations were also observed for PCDD/F and BDE-153 in mother’s milk lipids. Associations between tetra-pentaPBDEs and THs were not statistically significant. After covariate adjustment, the associations remained significant for PCDD/F TEQ and BDE-153 (Table 3). There were no outliers in the adjusted analysis of association between PCDD/F TEQ and total T3. When three outliers were excluded from the PBDE dataset, the association between BDE-153 and total T3 did not change considerably (β = −0.25, SE = 0.07, *p* = 0.001). In analyses restricted to individuals with data on PCDD/F, adjusted associations between total T3 and di-*ortho* PCB and mono-*ortho* PCB TEQ were still non-significant (results not shown). The association between total T3 and BDE-153 remained significantly inverse (β = −0.35, *p* = 0.006). There were no significant associations between PCDD/Fs, PCBs and PBDEs in serum or mother’s milk lipids and first trimester maternal serum concentrations of free T4 or TSH (Table 3).

**Table 3:** Regression coefficients (β, mean ± standard error) for associations between maternal body burdens of PCB, PCDD/F and PBDE and first trimester thyroid hormone concentrations and third trimester thyroid peroxidase antibody (a-TPO) concentrations

Body burdens^a^	*N*	Total T3	Free T4	TSH	a-TPO^b^
*Unadjusted model*					
di-*ortho* PCB	219	−0.15 ± 0.06*	0.04 ± 0.25	0.08 ± 0.15	0.013 ± 0.063
mono-*ortho* PCB TEQ	219	−0.15 ± 0.06*	0.16 ± 0.22	0.07 ± 0.14	−0.0096 ± 0.055
PCDD/F TEQ	67	−0.55 ± 0.14*	0.10 ± 0.67	0.45 ± 0.38	0.0055 ± 0.17
tetra-pentaPBDE	127	0.009 ± 0.06	0.35 ± 0.26	0.05 ± 0.16	0.035 ± 0.051
BDE-153	127	−0.25 ± 0.07*	0.01 ± 0.34	0.15 ± 0.20	0.072 ± 0.068
*Multivariate model* ^*c*^					
di-*ortho* PCB	218	−0.07 ± 0.09	−0.19 ± 0.34	0.08 ± 0.21	−0.28 ± 0.089
mono-*ortho* PCB TEQ	218	−0.10 ± 0.07	0.09 ± 0.27	0.003 ± 0.17	−0.029 ± 0.065
PCDD/F TEQ	66	−0.54 ± 0.21*	−0.43 ± 0.89	−0.01 ± 0.53	−0.18 ± 0.25
tetra-pentaPBDE	126	0.02 ± 0.06	−0.01 ± 0.21	0.14 ± 0.12	0.048 ± 0.052
BDE-153	126	−0.20 ± 0.08*	−0.34 ± 0.37	0.17 ± 0.22	0.085 ± 0.076

^a^Ln-transformed blood serum concentrations in late pregnancy; di-*ortho* PCB (sum of PCB 138, 153 and 180) and mono-*ortho* PCB TEQ (sum of TEQs for PCB 105, 118, 156 and 167) [32]. Ln-transformed mother’s milk concentrations of PCDD/F TEQ (sum of TEQs for the 17 congeners with WHO TEFs) [32], tetra-pentaPBDE (sum of BDE-47, BDE-99, BDE-100) and BDE-153

^b^a-TPO data were ln-transformed in the statistical analyses due to a log-normal distribution

^c^Associations adjusted for maternal age, pre-pregnancy BMI, smoking during pregnancy and season of sampling, *p ≤ 0.05

No associations were found between maternal PCDD/F, PCB and PBDE concentrations in serum or mother’s milk lipids and concentrations of anti-TPO in late pregnancy (Table 3). Furthermore, no significant associations between maternal PBDE concentrations in mother’s milk lipids and third trimester TH concentrations were found (Table 4). Finally, there were no significant associations between maternal PBDE concentrations in mother’s milk and THs in infants at 3 weeks and 3 months of age (Table 5).

**Table 4:** Regression coefficients (β, mean ± standard error) for associations between maternal body burdens of PBDEs and third trimester concentrations of thyroid hormones

Body burdens^a^	Total T3	Free T4	TSH
*Unadjusted model*			
tetra-pentaPBDE	0.03 ± 0.05	0.22 ± 0.11	−0.13 ± 0.09
BDE-153	−0.06 ± 0.06	0.08 ± 0.16	0.04 ± 0.12
*Multivariate model* ^*b*^			
tetra-pentaPBDE	−0.005 ± 0.04	0.19 ± 0.11	−0.06 ± 0.08
BDE-153	0.03 ± 0.06	−0.02 ± 0.16	−0.03 ± 0.13

^a^Ln-transformed mother’s milk concentrations of tetra-pentaPBDE (sum of BDE-47, BDE-99 and BDE-100) and BDE-153 (*N* = 166)

^b^Associations adjusted for maternal age, pre-pregnancy BMI, smoking during pregnancy, season of sampling and weight gain during pregnancy. *p ≤ 0.05

**Table 5:** Regression coefficients (β, mean ± standard error) for associations between maternal body burdens of PBDEs and concentrations of thyroid hormones in infants at 3 weeks and 3 months of age

Body burden^a^	3 weeks			3 months		
	Total T3	Free T4	TSH	Total T3	Free T4	TSH
Unadjusted model						
tetra-pentaPBDE	0.01 ± 0.03	−0.20 ± 0.28	0.004 ± 0.04	0.02 ± 0.02	0.14 ± 0.29	0.02 ± 0.04
BDE-153	0.03 ± 0.11	−0.36 ± 0.40	0.16 ± 0.19	0.14 ± 0.10	−0.33 ± 0.41	0.28 ± 0.17
Multivariate model^b^						
tetra-pentaPBDE	0.03 ± 0.03	−0.12 ± 0.30	0.02 ± 0.05	0.03 ± 0.03	−0.04 ± 0.30	0.03 ± 0.03
BDE-153	0.11 ± 0.11	−0.48 ± 0.42	0.01 ± 0.20	0.19 ± 0.11	−0.47 ± 0.44	0.15 ± 0.18

^a^Ln-transformed mother’s milk concentrations of tetra-pentaPBDE (sum of BDE-47, BDE-99 and BDE-100) and BDE-153 (*N* = 104-130)

^b^Associations adjusted for maternal age, pre-pregnancy BMI, smoking, alcohol consumption, infant’s sex and birth weight. *p ≤ 0.05

## Discussion

The POPUP cohort includes healthy Swedish mother-infant pairs exposed to relatively low background concentrations of POPs and with measured concentrations of thyroid hormones that were generally within reference intervals [33, 34]. In the present study we found significantly inverse associations between PCDD/F TEQ and BDE-153 concentrations in mother’s milk lipids and first trimester maternal concentrations of total T3 after covariate adjustment. Our findings may have implications for fetal development since it is dependent on maternal first trimester THs status, whereas the importance of fetal TH production becomes more apparent in the second trimester. The PCDD/Fs results for the first trimester corroborate with the results from the third trimester published in our previous study [24], strongly suggesting that the decreased total T3 with increasing PCDD/F body burden persisted during the whole pregnancy period.

Comparisons with results from other studies are hampered by differences in study design. We could not find other studies looking at PCDD/Fs and TH status at different time points during pregnancy. In a Dutch study significantly inverse associations were reported between PCDD/F and non/mono-*ortho* PCB concentrations in mother’s milk and maternal total T3 and total T4 in late pregnancy [9]. However, the results were not adjusted for potential confounders, as in our study. When maternal serum lipid dioxin-like PCBs concentrations were included together with the PCDD/Fs in the present study, using the WHO2005 TEF system [32], PCDD/F/PCB TEQs were not associated with total T3 during the first and third trimester (1st: β = −0.31 ± 0.22, *p* = 0.16, *N* = 64; 3rd: β = −0.15 ± 0.16, *p* = 0.35, *N* = 90). This corroborates with results from a cohort of pregnant women from Germany, where no confounder-adjusted association was found between serum or mother’s milk PCDD/F + dioxin-like PCB toxicity equivalent (TEQ) concentrations and TSH, total T4, free T4, total T3 and free T3 concentrations in late pregnancy [13].

We observed no significant associations between the hexa-pentaPBDEs (sum of BDE-47, −99, −100) and first and third trimester THs in the present study. Also in statistical analyses of individual BDE-47, −99 and −100 no significant associations were found with first and third trimester THs (results not shown), suggesting that tetra-pentaPBDEs do not markedly influence the TH status of pregnant women with background exposure in Sweden. Results from other studies of PBDE exposure and THs in pregnant women are difficult to compare because of large differences in study design and exposure levels. Among larger studies from North America the results vary significantly, with one study showing an inverse association between maternal serum lipid PBDE (sum of BDE-28, −47, −99, −100 and −153) and third trimester TSH concentrations, and three other studies reporting varying associations with T3 and T4 but no associations with TSH [21, 22, 35]. PBDE body burdens were about 10-fold higher among North American women than among our study participants.

Many of the previous PBDE studies have only investigated maternal TH status at one time point during pregnancy. However, Abdelouahab et al. [22] found a confounder-adjusted inverse association between maternal plasma lipid BDE-47 and −99 concentrations in US women and first trimester total T4, total T3, free T4 and free T3 (not BDE-99), but these associations did not persist at delivery except for BDE-47 and free T4. BDE-153 was not included as a single congener in the statistical analyses [22].

Chevrier [36] have discussed important factors to consider in studies of thyroid hormones and PBDEs that may be responsible for differences in results between studies. These factors include iodine status, method for lipid adjustment of PBDE concentrations in blood, method used for analysis of free TH, presence of hydroxylated PBDE metabolites and reverse causality [36]. We propose that in future studies a prospective approach should be used, with assessment of POP body burdens starting before pregnancy and with repeated measurement of maternal TH status during the whole study period. Ideally repeated measurements of maternal POP body burdens should also be included in order to determine if there are associations between changes in POP body burdens and changes in TH status. Inclusion of a broad battery of biochemical/functional markers of thyroid system function, as well as nutritional status (iodine, selenium, etc.), would improve possibilities of determining possible mechanisms of POP-induced disruption of the thyroid system. The same study design should be used on different cohorts around the world in order to improve conclusions about causality of associations found.

The inverse BDE-153 association with maternal first trimester total T3 observed by us did, in contrast to the PCDD/F association [24], not persist into the third trimester. There was no correlation between PCDD/F and BDE-153 concentrations in mother’s milk (Pearson’s *r* = 0.087, *p* = 0.41, *N* = 93). Nevertheless we investigated if associations with first trimester total T3 were changed in a regression model with both PCDD/F and BDE-153 included. In this case the BDE-153 association was strengthened and remained statistically significant (β = −0.28 ± 0.13, *p* = 0.035, *N* = 62), whereas the PCDD/F association was slightly weakened and became non-significant (β = −0.35 ± 0.23, *p* = 0.14; *N* = 62). Similarly the association between PCDD/F and third trimester, reported in Darnerud et al. [24] (β = −0.32 ± 0.16, *p* = 0.050, *N* = 91) was weakened and became non-significant after adjustment for BDE-153 (β = −0.30 ± 0.17, *p* = 0.087, *N* = 87). In this case the BDE-153 association with third trimester T3 remained non-significant (results not shown). The results suggest that although there was no significant correlation between mother’s milk lipid PCDD/F and BDE-153 concentrations, there may be some shared variance that explains some of the observed associations with total T3.

We can only speculate why there were differences in association between BDE-153 and total T3 in the first (inverse association) and third trimester (no association), when associations between PCDD/Fs and total T3 were more consistent (inverse at both occasions). Large physiological changes occur between first and third trimesters, and the demand on the maternal thyroid system most probably also changes. During the first trimester, fetal development is dependent on maternal thyroid status, whereas later in pregnancy fetal thyroid function takes over. It may be possible that there are first and third trimester differences in the capacity of the maternal thyroid system to compensate for POP disruption. In fact the association between PCDD/F and total T3 appeared to be stronger in early pregnancy than in later pregnancy, as suggested by a lower β coefficient for the association in the third trimester (β = −0.32) [24] than in the first trimester (β = −0.54) (present study).

Mother’s milk lipid concentrations of PCDD/Fs and PBDEs were used by us as a measure of maternal body burden during pregnancy, which makes it difficult to draw conclusions about causality of observed associations. Reverse causality may be possible since maternal T3 status could have influenced distribution, metabolism and excretion of the contaminants. For instance, mammary gland transfer of the contaminants from maternal blood into mother’s milk may be affected by TH status. Moreover, maternal serum lipid concentrations of the contaminants may be affected, thus indirectly influencing the mother’s milk concentrations. We could not find published studies on influences of maternal TH status on mammary gland PCDD/F and PBDE transfer. Several observations do however suggest that TH status does not influence transfer of POPs to mother’s milk, such as a positive correlation (Pearson’s *r* = 0.98) between serum lipid and mother’s milk lipid BDE-153 concentrations 3 weeks after delivery in the POPUP cohort [37], and a positive correlation between maternal third trimester serum lipid di-*ortho* PCB concentrations and mothers milk lipid di-*ortho* PCB concentrations (Pearson’s *r* = 0.93; *N* = 202) among POPUP mothers with both matrices analysed. Transfer of lipid soluble PCDD/Fs, PCBs and PBDEs from maternal blood to mother’s milk is most likely a passive process, although it varies depending on the lipid solubility of the contaminants, number of chlorines in the molecule, and molecular weight and volume [38]. The possibility the maternal TH status influences maternal serum lipid POP concentrations is not supported by the lack of associations between maternal first (the present study) and third trimester [24] TH status and maternal serum lipid mono- and di-*ortho* PCB concentrations. Moreover, a strong positive intra-individual correlation in di-*ortho* PCB concentrations during pregnancy has been found [39, 40], suggesting that physiological changes during pregnancy do not bias serum lipid POP concentrations in late pregnancy compared to concentrations before pregnancy or in early pregnancy.

The mean decrease in first trimester concentrations of total T3 was 0.54 nmol/L (95 % confidence interval: 0.13-0.95) for every 1-unit increase in ln-transformed mother’s milk lipid PCDD/F TEQ concentration. This corresponds to a mean difference in total T3 of approximately 0.3 nmol/L between the 25th (5.8 pg TEQ/g lipid) and 75th (9.7 pg TEQ/g lipid) percentile of PCDD/F TEQ body burdens. The median first trimester total T3 concentration was 2.4 nmol/L, and total T3 normally is relatively stable over time [41]. Consequently our observed PCDD/F-induced change, if causal, could be viewed as a significant shift in total T3 status during the first trimester when the fetus is dependent on maternal TH status. It may however be argued that it would be more relevant to measure free T3 concentrations in serum than total T3, since free T3 is the biologically available fraction of total T3. In the previous study when THs in late pregnancy were analysed, total T3, free T4 and TSH were regarded as the most relevant THs to measure [24], since a method for measuring free T3 was not available in the laboratory. This was also the case a few years later when THs in early pregnancy were analysed.

The developing fetus is dependent on maternal thyroid status during the first 20 weeks of gestation [36]. Our findings of inverse associations between maternal PCDD/F or PBDE-153 body burdens and circulating total T3 in week 6–12 may have implications for normal fetal development. Recent studies show that extracellular T3 may act via pathways mediated by for instance integrin hormone receptor domains in the apical membranes of many types of cells [42]. Further studies of possible involvement of T3 in regulation of physiological function through such extracellular pathways are needed before conclusions may be drawn about the significance of PCDD/F- and PBDE-induced alterations in T3 status for human health.

In the present investigation, no significant associations were observed between prenatal exposure to PBDEs and infant concentrations of THs [24]. Among other studies on PBDE exposure and infant TH concentrations some studies have been small or have reported unadjusted correlations [43–47]. Larger studies, taking possible confounding of PBDE and TH associations into account [14, 17–20], have reported inverse, positive, as well as no significant associations between PBDE exposure and THs in infant serum or cord blood. This further illustrates the difficulties in interpreting the significance of the findings reported in POP/TH research [36].

It could be argued that a large portion of the PBDE exposure of infants occurs after birth through breast feeding. To assess if this route of PBDE exposure may have influenced infant TH status at 3 months of age we estimated mother’s milk exposure by multiplying PBDE concentrations in mother’s milk (ng/g fresh weight) with number of days of full breast feeding during the first 3 months of life. In this case a day with reported partial breastfeeding was accounted for as ½ day of full feeding. We did not observe significant associations between mother’s milk exposure to tetra-pentaPBDE and TSH (β = 0.00053 ± 0.0029; mean ± SE; *p* = 0.85; *N* = 101), free T4 (β = −0.013 ± 0.020; *p* = 0.54; *N* = 101) and total T3 (β = −0.00047 ± 0.0020; *p* = 0.79; *N* = 101), after adjusting for maternal age, maternal pre-pregnancy BMI, maternal smoking, maternal alcohol consumption, infant sex and birth weight. Similarly no associations were observed between mother’s milk exposure to BDE-153 and TSH (β = −0.023 ± 0.028; *p* = 0.41; *N* = 101), free T4 (β = −0.19 ± 0.20; *p* = 0.36; *N* = 101) and free T4 (β = −0.027 ± 0.017; *p* = 0.12; *N* = 101). Similarly, in the previous study we did not observe significant associations between mother’s milk exposure to PCDD/Fs and PCBs and infant TH status at 3 months of age [24].

We did not find any significant associations between PCDD/F, PCB and PBDE body burdens and third trimester maternal serum anti-TPO concentrations, suggesting that the POPs at background concentrations in Sweden do not induce thyroid dysfunction in pregnant women by the anti-TPO mechanisms. Our finding of no associations with PCBs is in agreement with a study of adults in Italy [27]. In a study of adults living in a highly PCB polluted area and in two less polluted areas of Slovakia, the highly exposed population had a higher frequency of a positive test for anti-TPO [26].

The main strengths of the present study are the relatively large sample size, the adjustment of results for possible confounding, and the availability of maternal and infant TH from two time periods. However, we cannot exclude the possibility that the observed associations with PCDD/Fs and BDE-153 may be a result of residual confounding. For example, we did not have data on iodine and selenium status of the participating mothers. Moreover, because of the cross-sectional design of the study, it is not possible to draw conclusions about causality of the PCDD/F and BDE-153 results, although the lack of associations for PCBs and other PBDEs suggests that maternal TH status in general does not affect serum and mother’s milk concentrations of lipid soluble POPs.

## Conclusions

We found that higher maternal body burdens of PCDD/Fs and BDE-153 were associated with lower concentrations of first trimester total T3 in this Swedish cohort. The present study shows that BDE-association did not persist into the third trimester, whereas our previous study shows that the PCDD/F association does [24]. Taking both the PBDE results in the present study and the PCDD/F results in the previous study into account, the observed maternal associations were not mirrored in the infants, suggesting that POP-induced changes in maternal TH status do not necessarily result in significant changes in infant TH status. The large variation in study design of previously published studies makes it difficult to compare results. In order to reach more certain conclusions about causality of associations between POP-exposure and maternal/infant TH status, prospective studies with repeated measurements of a wide range of biochemical/functional markers of TH status and POPs before, during and after pregnancy should be performed on maternal/infant cohorts from different parts of the world.

## Abbreviations

anti-TPO: Antibodies to thyroid peroxidase
BDE: Brominated diphenyl ether
BMI: Body mass index
LOQ: Limit of quantification
PBDE: Polybrominated diphenyl ether
PCB: Polychlorinated biphenyl
PCDD: Polychlorinated dibenzo-p-dioxin
PCDF: Polychlorinated dibenzofuran
POP: Persistent organic pollutant
POPUP: Persistent organic pollutants in Uppsala primiparas
T3: Triiodo-thyronine
T4: Thyroxine
TEF: Toxicity equivalent factor
TEQ: Toxicity equivalent
TH: Thyroid hormone
TSH: Thyroid stimulating hormone

## References

[1] Williams GR (2008). Neurodevelopmental and neurophysiological actions of thyroid hormone. J Neuroendocrinol..

[2] Trumpff C, De Schepper J, Tafforeau J, Van Oyen H, Vanderfaielle J, Vandevijvere S (2013). Mild iodine deficiency in pregnancy in Europe and its consequences for cognitive and psychomotor development of children: a review. J Trace Elem Med Biol..

[3] Colicchia M, Campagnolo L, Baldini E, Ulisse S, Valensise H, Moretti C (2014). Molecular basis of thyrotropin and thyroid hormone action during implantation and early development. Hum Reprod Update..

[4] Boas M, Main KM, Feldt-Rasmussen U (2009). Environmental chemicals and thyroid function: an update. Curr Opin Endocrinol Diabetes Obes..

[5] Forhead AJ, Fowden AL (2014). Thyroid hormones in fetal growth and prepartum maturation. J Endocrinol..

[6] Brouwer A, Morse DC, Lans MC, Schuur AG, Murk AJ, Klasson-Wehler E, Bergam A, Visser TJ (1998). Interactions of persistent environmental organohalogens with the thyroid hormone system: mechanisms and possible consequences for animal and human health. Toxicol Ind Health..

[7] Chevrier J, Eskenazi B, Bradman A, Fenster L, Barr DB (2007). Associations between prenatal exposure to polychlorinated biphenyls and neonatal thyroid-stimulating hormone levels in a Mexican-American population, Salinas Valley, California. Environ Health Perspect..

[8] Chevrier J, Eskenazi B, Holland N, Bradman A, Barr DB (2008). Effects of exposure to polychlorinated biphenyls and organochlorine pesticides on thyroid function during pregnancy. Am J Epidemiol..

[9] Koopman-Esseboom C, Morse DC, Weisglas-Kuperus N, Lutkeschipholt IJ, der Paaw V, Tuinstra LG, Brouwer A, Sauer PJ (1994). Effects of dioxins and polychlorinated biphenyls on thyroid hormone status of pregnant women and their infants. Pediatr Res..

[10] Takser L, Mergler D, Baldwin M, De G, Smargiassi A, Lafond J (2005). Thyroid hormones in pregnancy in relation to environmental exposure to organochlorine compounds and mercury. Environ Health Perspect.

[11] Wang SL, Su PH, Jong SB, Guo YL, Chou YL, Chou WL, Päpke O (2005). In utero exposure to dioxins and polychlorinated biphenyls and its relations to thyroid function and growth hormone in newborns. Environ Health Perspect..

[12] Dallaire R, Dewially E, Ayotte P, Muckle G, Laliberté C, Bruneau S (2008). Effects of prenatal exposure to organochlorines on thyroid hormone status in newborns from two remote coastal regions in Quebec, Canada. Environ Res..

[13] Wilhelm M, Wittsiepe J, Lemm F, Ranft U, Krämer U, Fürst P, Röseler SC, Greshake M, Imöhl M, Eberwein G, Rauchfuss K, Kraft M, Winneke G (2008). The Duisburg birth cohort study: influence of the prenatal exposure to PCDD/Fs and dioxin-like PCBs on thyroid hormone status in newborns and neurodevelopment of infants until the age of 24 months. Mutat Res..

[14] Herbstman JB, Sjödin A, Apelberg BJ, Witter FR, Halden RU, Patterson DG, Panny SR, Needham LL, Goldman LR (2008). Birth delivery mode modifies the associations between prenatal polychlorinated biphenyl (PCB) and polybrominated diphenyl ether (PBDE) and neonatal thyroid hormone levels. Environ Health Perspect..

[15] Hisada A, Shimodaira K, Okai T, Watanabe K, Takemori H, Takasuga T, Noda Y, Shirakawa M, Kato M, Yoshinaga J (2013). Serum levels of hydroxylated PCBs, PCBs and thyroid hormone measures of Japanese pregnant women. Environ Health Prev Med..

[16] Chevrier J, Harley KG, Bradman A, Sjödin A, Eskenazi B (2010). Polybrominated diphenyl ether (PBDE) flame retardants and thyroid hormone during pregnancy. Environ Health Perspect..

[17] Eggesbø M, Thomsen C, Jørgensen JV, Becher G, Odland JØ, Longnecker MP (2011). Associations between brominated flame retardants in human milk and thyroid-stimulating hormone (TSH) in neonates. Environ Res..

[18] Chevrier J, Harley KG, Bradman A, Sjödin A, Eskenazi B (2011). Prenatal exposure to polybrominated diphenyl ether flame retardants and neonatal thyroid-stimulating hormone levels in the CHAMACOS study. Am J Epidemiol..

[19] Lin SM, Chen FA, Huang YF, Hsing LL, Chen LL, Wu LS, Liu TS, Chang-Chien GP, Chen KC, Chao HR (2011). Negative associations between PBDE levels and thyroid hormones in cord blood. Int J Hyg Environ Health..

[20] Shy CG, Huang HL, Chao HR, Chang-Chien GP (2012). Cord blood levels of thyroid hormones and IGF-1 weakly correlate with breast milk levels of PBDEs in Taiwan. Int J Hyg Environ Health..

[21] Stapleton HM, Eagle S, Anthopolos R, Wolkin A, Miranda ML (2011). Associations between polybrominated diphenyl ether (PBDE) flame retardants, phenolic metabolites, and thyroid hormones during pregnancy. Environ Health Perspect..

[22] Abdelouahab N, Langlois MF, Lavoie L, Corbin F, Pasquier JC, Takser L (2013). Maternal and cord-blood thyroid hormone levels and exposure to polybrominated diphenyl ethers and polychlorinated biphenyls during early pregnancy. Am J Epidemiol..

[23] Dallaire R, Muckle G, Dewailly E, Jacobson SW, Jacobson JL, Sandanger TM, Sandau CD, Ayotte P (2009). Thyroid hormone levels of pregnant inuit women and their infants exposed to environmental contaminants. Environ Health Perspect..

[24] Darnerud PO, Lignell S, Glynn A, Aune M, Törnkvist A, Stridsberg M (2010). POP levels in breast milk and maternal serum and thyroid hormone levels in mother-child pairs from Uppsala, Sweden. Environ Int..

[25] Lignell S, Aune M, Darnerud PO, Hanberg A, Larsson SC, Glynn A (2013). Prenatal exposure to polychlorinated biphenyls (PCBs) and polybrominated diphenyl ethers (PBDEs) may influence birth weight among infants in a Swedish cohort with background exposure: a cross-sectional study. Environ Health..

[26] Langer P, Kocan A, Taitáková M, Susienková K, Rádiková Z, Koska J, Ksinantová L, Imrich R, Hucková M, Drobná B, Gasperiková D, Trnovec T, Klimes I (2009). Multiple adverse thyroid and metabolic health signs in the population from the area heavily polluted by organochlorine cocktail (PCB, DDE, HCB, dioxin). Thyroid Res..

[27] Donato F, Zani C, Magoni M, Gelatti U, Covolo L, Orizio G, Speziani F, Indelicato A, Scarcella C, Bergonozi R, Apostoli P (2008). Polychlorinated biphenyls and thyroid hormone serum concentrations among people living in a highly polluted area: a cross-sectional population-based study. Environ Res..

[28] Lignell S, Aune M, Darnerud PO, Cnattingius S, Glynn A (2009). Persistent organochlorine and organobromine compounds in mother’s milk from Sweden 1996–2006: compound-specific temporal trends. Environ Res..

[29] Lignell S, Aune M, Darnerud PO, Soeria-Atmadja D, Hanberg A, Larsson S, Glynn A (2011). Large variation in breast milk levels of organohalogenated compounds is dependent on mother’s age, changes in body composition and exposures early in life. J Environ Monit..

[30] Glynn A, Aune M, Darnerud PO, Cnattingius S, Bjerselius R, Becker W, Lignell S (2007). Determinants of serum concentrations of organochlorine compounds in Swedish pregnant women: a cross-sectional study. Environ Health..

[31] Van der Velde EG, Marsman JA, de Jong APJM, Hoogerbrugge R, Liem AKD (1994). Analysis and occurrence of toxic planar PCBs, PCDDs and PCDFs in milk by use of carbosphere activated carbon. Chemosphere.

[32] Van den Berg M, Birnbaum LS, Denison M, De Vito M, Farland W, Feely M, Fielder H, Hakansson H, Hanberg A, Haws L, Rose M, Safe S, Schrenk D, Tohyama C, Trischer A, Tuomisto J, Tysklind M, Walker N, Peterson RE (2006). The 2005 World Health Organization reevaluation of human and Mammalian toxic equivalency factors for dioxins and dioxin-like compounds. Toxicol Sci..

[33] Elmlinger MW, Kühnel W, Lambrecht HG, Ranke MB (2001). Reference intervals from birth to adulthood for serum thyroxine (T4), triiodothyronine (T3), free T3, free T4, thyroxine binding globulin (TBG) and thyrotropin (TSH). Clin Chem Lab Med..

[34] Stricker R, Echenard M, Eberhart R, Chevailler MC, Perez V, Quinn FA, Stricker R (2007). Evaluation of maternal thyroid function during pregnancy: the importance of using gestational age-specific reference intervals. Eur J Endocrinol..

[35] Vuong AM, Webster GM, Romano ME, Braun JM, Zoeller RT, Hoofnagle AN, Sjödin A, Yolton K, Lanphear BP, Chen A (2015). Maternal Polybrominated Diphenyl Ether (PBDE) Exposure and Thyroid Hormones in Maternal and Cord Sera: The HOME Study, Cincinnati, USA. Environ Health Perspect.

[36] Chevrier J (2013). Invited commentary: Maternal plasma polybrominated diphenyl ethers and thyroid hormones--challenges and opportunities. Am J Epidemiol..

[37] Darnerud PO, Lignell S, Aune M, Isaksson M, Cantillana T, Redeby J, Glynn A (2015). Time trends of polybrominated diphenylether (PBDE) congeners in serum of Swedish mothers and comparisons to breast milk data. Environ Res..

[38] Mannetje A, Coakley J, Mueller JF, Harden F, Toms LM, Douwes J (2012). Partitioning of persistent organic pollutants (POPs) between human serum and breast milk: a literature review. Chemosphere..

[39] Glynn A, Larsdotter M, Aune M, Darnerud PO, Bjerselius R, Bergman Å (2011). Changes in serum concentrations of polychlorinated biphenyls (PCBs), hydroxylated PCB metabolites and pentachlorophenol during pregnancy. Chemosphere..

[40] Longnecker MP, Klebanoff MA, Gladen BC, Berendes HW (1999). Serial levels of serum organochlorines during pregnancy and postpartum. Arch Environ Health..

[41] Abdalla SM, Bianco AC (2014). Defending plasma T3 is a biological priority. Clin Endocrinol (Oxf).

[42] Cheng SY, Leonard JL, Davis PJ (2010). Molecular aspects of thyroid hormone actions. Endocr Rev..

[43] Kim TH, Lee YJ, Lee E, Patra N, Lee J, Kwack SJ, Kim KB, Chung KK, Han SY, Han JY, Lee BM, Kim HS (2009). Exposure assessment of polybrominated diphenyl ethers (PBDE) in umbilical cord blood of Korean infants. J Toxicol Environ Health A..

[44] Roze E, Maijer L, Bakker A, Van Braeckel KN, Sauer PJ, Bos AF (2009). Prenatal exposure to organohalogens, including brominated flame retardants, influences motor, cognitive, and behavioral performance at school age. Environ Health Perspect..

[45] Mazdai A, Dodder NG, Abernathy MP, Hites RA, Bigsby RM (2003). Polybrominated diphenyl ethers in maternal and fetal blood samples. Environ Health Perspect..

[46] Kim TH, du Bang Y, Lim HJ, Won AJ, Ahn MY, Patra N, Chung KK, Kwack SJ, Park KL, Han SY, Choi WS, Han JY, Lee BM, Oh JE, Yoon JH, Lee J, Kim HS (2012). Comparisons of polybrominated diphenyl ethers levels in paired South Korean cord blood, maternal blood, and breast milk samples. Chemosphere..

[47] Kim UJ, Lee IS, Kim HS, Oh JE (2011). Monitoring of PBDEs concentration in umbilical cord blood and breast milk from Korean population and estimating the effects of various parameters on accumulation in humans. Chemosphere..

